# Modelling the optimization of world-class 400 m and 1,500 m running performances using high-resolution data

**DOI:** 10.3389/fspor.2024.1293145

**Published:** 2024-03-05

**Authors:** Antoine Le Hyaric, Amandine Aftalion, Brian Hanley

**Affiliations:** ^1^ Laboratoire Jacques-Louis Lions (LJLL), CNRS, Inria, Sorbonne Université, Université de Paris, Paris, France; ^2^ Centre D’Analyse et de Mathématique Sociales, CNRS UMR-8557, Ecole des Hautes Etudes en Sciences Sociales, Paris, France; ^3^ Carnegie School of Sport, Leeds Beckett University, Leeds, United Kingdom

**Keywords:** athletics, bends, coaching, pacing, track and field

## Abstract

The 400 m and 1,500 m are track events that rely on different but important contributions from both the aerobic and anaerobic energy systems. The purpose of this study is to model men's and women's 400 m and 1,500 m championship performances to gain a deeper understanding of the key mechanical and physiological factors affecting running speed and bend running using high-resolution data from live competition (10 Hz). To investigate World-class athletes' instantaneous speeds, propulsive forces and aerobic and anaerobic energy, we model and simulate the performances of the men's and women's European Athletics 400 m champions, Matthew Hudson–Smith and Femke Bol, as well as the men's European Athletics 1,500 m champion, Jakob Ingebrigtsen, and the women's European Athletics U23 1,500 m champion, Gaia Sabbatini. The simulations show that a fast start is essential in both the 400 m and 1,500 m because of the need for fast oxygen kinetics, with peak running speeds occurring within the first ∼50 m in both events. Subsequently, 400 m athletes slow continually from this maximum speed to the finish, and a total anaerobic contribution of ∼77% is found for both male and female champions. The key to faster 400 m racing is to reduce the decrease in velocity: this comes from both a high VO_2_ and a high anaerobic contribution. Ingebrigtsen's winning tactic in the European 1,500 m final is to adopt a very fast cruising pace from 300 m onwards that is possible because he is able to maintain a high VO_2_ value until the end of the race and has a large anaerobic contribution. He has fast VO_2_ kinetics that does not require as fast a start as his opponents, but then he speeds up in the last two laps, without a fast sprint finish. The comparison between Sabbatini's slower and quicker races (∼8 s difference) shows that it is the improvement of aerobic metabolism that has the greatest effect on 1,500 m performance. Coaches should note in particular that the all-out pacing nature of the 400 m requires the prioritization of anaerobic energy system development, and those who coach the 1,500 m should note the differing energy contributions between even-paced races and championship racing.

## 1 Introduction

The issue of determining the optimal pacing strategy in a race is a crucial one. Whatever the distance, there is no consensus on the best pacing strategy (1, 2) or proportions of aerobic/anaerobic energy contributions (3, 4). Since it is not possible to undertake physiological measures *in vivo* during a race to determine the instantaneous contributions of aerobic and anaerobic energy, a study that models the mechanical and physiological factors affecting performance and speed can provide a richer insight than using time splits or experimental measures. To investigate World-class athletes' instantaneous speeds, propulsive force, aerobic and anaerobic energy, we use high-resolution data from live competition to model the effect of performance factors in the 400 m and 1,500 m. These two races correspond to two different types of pacing strategies: the shorter event being a sprint where athletes use an all-out strategy and decelerate in the second half of the race (5), whereas championship 1,500 m racing features variable pacing with considerable acceleration over the last 300 m (6).

The outdoor 400 m race is one complete lap of an athletics track and comprises two straight sections and two bends; on a standard track, the bends are 116 m long, and the straights 84 m long (7). Coaches have been advised to pay attention to the potentially negative biomechanical effects of certain lanes (8), and the effect of running on bends has been analyzed for sprint races (9–14). However, their effects on the 400 m, where the whole race is run in lanes, and on the 1,500 m, where seven bends are negotiated but not in lanes, are not yet fully explained. In addition, the short duration of the 400 m, with most male and female Olympic 2020 finalists achieving finishing times <45 s and <51 s, respectively (15), means it relies more on anaerobic energy than aerobic resources for total energy requirements (16). To date, research on pacing in the 400 m event has relied on 50-m or 100-m splits [e.g. (5, 17),] and has shown a rapid acceleration in the first 100–150 m, with a gradual decrease in speed until the finish (18, 19). For this reason, the 400 m sprint is often classed by coaches instead as a “speed endurance” event (20), where the ability to maintain speed despite the depletion of anaerobic reserves is a key performance indicator. With most research on pacing in the 1,500 m using split times only every 100 m at best (2, 21), obtaining accurate measurements of instantaneous velocity that give more precise values for maximum velocity, the effect of bend running, and the sprint finish has not previously been possible.

In the present study, rather than using statistical analyses of 100-m split times or big data, we choose to analyze a select sample of World-class athletes individually using high-resolution data and fit a mathematical model to their pacing profiles. This process gives access to modeled instantaneous velocity, propulsive force, anaerobic energy and VO_2_ throughout the race and thus helps us explain the parameters that influence race strategy or the ability to optimize running speed. This model has been previously used to analyze the 10,000 m (22) but, for that study, only time splits every 100 m were available rather than instantaneous values of running speed. In addition, using models that simulate an athlete's instantaneous velocity and allow for the manipulation of contributing variables (e.g., energy, propulsive force) will improve our understanding of performances in 400 m and 1,500 m racing and inform coaches of appropriate training practices and tactical possibilities. Using such a deterministic model means that new specific computations are involved for each race and each athlete, and so in this study we focus on the performances of specific World-class athletes in certain races for which sufficiently high quality data are available. The aim of this study is to model World-class 400 m and 1,500 m racing performances to allow for a deeper understanding of the factors that affect running speed and to establish the effect of the bends on performance.

## 2 Materials and methods

In this study, we analyze several 400 m and 1,500 m race data that were recorded by Matsport, who control the data, using IsoLynx technology during the European Athletics Championships in Munich (GER) in 2022 and the European Athletics U23 Championships in Tallinn (EST) in 2021. The IsoLynx Real-Time Location System (RTLS) athlete tracking system uses a network of wireless athlete tags (in the name/number bibs) to capture live in-race data including velocity, acceleration, and distance traveled. Each chip provides data to the system, feeding the “filter engine”, which generates a chip location algorithm as output. The interval for this data output is typically 100 ms and it is this sampling rate (10 Hz), used in broadcasts, that we use in this paper to analyze instantaneous velocity for each runner.

Subsequently, we fit a mathematical model onto these velocity data for any selected athlete. This mathematical model allows us to perform further predictive simulations and analyze the effect of different physiological variables on performance. We also predict what values the athlete had for particularly important variables (e.g., anaerobic reserve, peak aerobic value). The resulting modeled data can then be manipulated for individual variables to ascertain their effects (by increasing or decreasing the values, for instance).

### 2.1 Description of the track

Both the 2022 European Athletics Championships and 2021 European Athletics U23 Championships were held on standard athletics tracks that were 400 m long, made up of straights of 84 m and half circles of radius 36.5 m, acknowledging that the length of the track (in the inside lane) is measured 0.30 m from the kerb so that the curvature of the track is either zero (in the straights) or 1/36.5 (in the bends) (7). The 400 m race begins on the first bend using a staggered start, and then the 116-m length of the bends and the 84-m length of the straights alternate. The 1,500 m race begins on a curved line at the start of the back straight.

From the 2022 European Athletics Championships in Munich, we present the simulations of the women's and men's 400 m winners: Femke Bol (NED) and Matthew Hudson-Smith (GBR), respectively. Also from the 2022 European Athletics Championships, we present the simulation of the men's 1,500 m winner, Jakob Ingebrigtsen (NOR). We compare Ingebrigtsen's race in Munich with his performances in Eugene (2022 World Championships, 2nd place) (23) and Chorzów [2023 Diamond League, personal record (PR)] (24). We model two performances for the same female 1,500 m athlete, Gaia Sabbatini (ITA): one from the European U23 Championships in Tallinn in 2021 (1st place, finishing time: 4:13.98) and the other from the 2022 European Athletics Championships in Munich in 2022 (9th place, finishing time: 4:06.04). These performances were chosen because they allow us to show, using different athletes as examples, the effects of the lane (on 400 m racing), the effects of the bends and performance parameters (both events), and comparisons between performances by the same athletes (1,500 m racing). Athlete data are used as a template for pacing onto which we superimpose simulations that can be manipulated. The original modeled data follow the original data obtained, but effectively correct for noise, and we can use these to explain the athlete's performance in the analyzed race. For example, the model indicates the total aerobic and anaerobic contributions that are calculated to have been used to achieve the performances recorded, as well as propulsive force and motor control.

### 2.2 Deterministic model

The process of running involves a control phenomenon in the human body; a runner who varies his speed has to modify his effort. The issue is how to model mathematically this neuro-muscular and energetical process controlling human effort. The model (9–11, 22, 25) yields an optimal control problem based on a system of coupled ordinary differential equations for the instantaneous velocity *v(s)*, the propulsive force per unit of mass *f(s)*, and the anaerobic energy *e(s),* where *s* is the distance from the start. The system relies on Newton's second law of motion (mechanics), on an equation for the variation in neural drive (motor control), and the energy balance that takes into account the aerobic contribution VO_2_, the anaerobic contribution *e(s)*, and the power developed by the propulsive force. The energy equation is an improvement on Morton's hydraulic analogy (26), as explained in Aftalion and Bonnans (25).

Simulations require numerical values for the athletes' parameters. These parameters are not measured, but as we explain below, are identified to match the data. The key physiological parameters that influence pacing are:

• the maximal propulsive force per unit of mass, *f_M_*.
• the global friction coefficient, *τ*, that encompasses all kinds of friction, both from joints and track and is linked to the runner's economy. This variable is less important for 400 m racing.
• the maximal decrease rate and increase rate of the propulsive force, which is related to motor control: an athlete cannot stop or start his or her effort instantaneously, but needs some time or distance to do so.
• the total anaerobic energy or maximal accumulated oxygen deficit, *e^0^*.
• the VO_2_ profile as a function of distance: because it is estimated that one liter of oxygen produces energy of about 21*.*1 kJ via aerobic cellular mechanisms (27), the energetic equivalent of a VO_2_ of X ml/min/kg is X × 21*.*1 kJ/min/kg so VO_2_ and aerobic energy expenditure are proportional. The VO_2_ increases during sprint events. For middle-distance races, the profile has three parts: an increase to the mean race value, which is then maintained for some distance, and then a decrease at the end of the race. This has been previously measured and analyzed (18, 21) and included in our model. Thus, there are parameters *γ*_1_ and *γ*_2_ that characterize the changes of behavior. These parameters do not depend on distance, but on residual anaerobic energy. We refer the reader to Aftalion et al. (9–11) for more details of the model.

The parameters *e^0^*, *f_M_*, *τ,* *γ*_1_ and *γ*_2_ and the maximal and final VO_2_ are not measured for any individual athlete (e.g., using experimental methods), but are estimated through a computation to fit the data. More precisely, they are identified for a specific race and athlete. For fixed values of the parameters, the optimal control problem is solved using the Ipopt interior point algorithm (28). It requires Jacobian and Hessian matrices, which are evaluated through automatic differentiation by the CasADi framework (29). Then, finding the best parameter values for each particular athlete and race uses the particle swarm optimization method from the PySwarms library (30). In total, we minimize the error between a single optimization simulation and data for a wide range of parameters to identify those that best match the data. A crucial piece of information included in our model is the centrifugal force on the bends; it does not act as such in the equation of motion but limits the propulsive force, *f(s)*, since it yields a decrease in the effective propulsive force on the bends:

$$f(s{)}^{2}+\frac{v{\left(s\right)}^{4}}{R{\left(s\right)}^{2}}\leq f_{tot}^{2}$$

where *R(s)* is the radius of curvature at distance *s* from the start and *f*_tot_ is the total force that the runner can exert, which also encompasses an effort to lean inwards or straighten at the beginning and end of the bends, respectively. For this purpose, we use the neural drive equation of Le Bouc et al. (31) for both *f* and *f*_tot_, which provides a very accurate description of bend running.

## 3 Results

### 3.1 Description of the 400 m races

**Figure 1:**
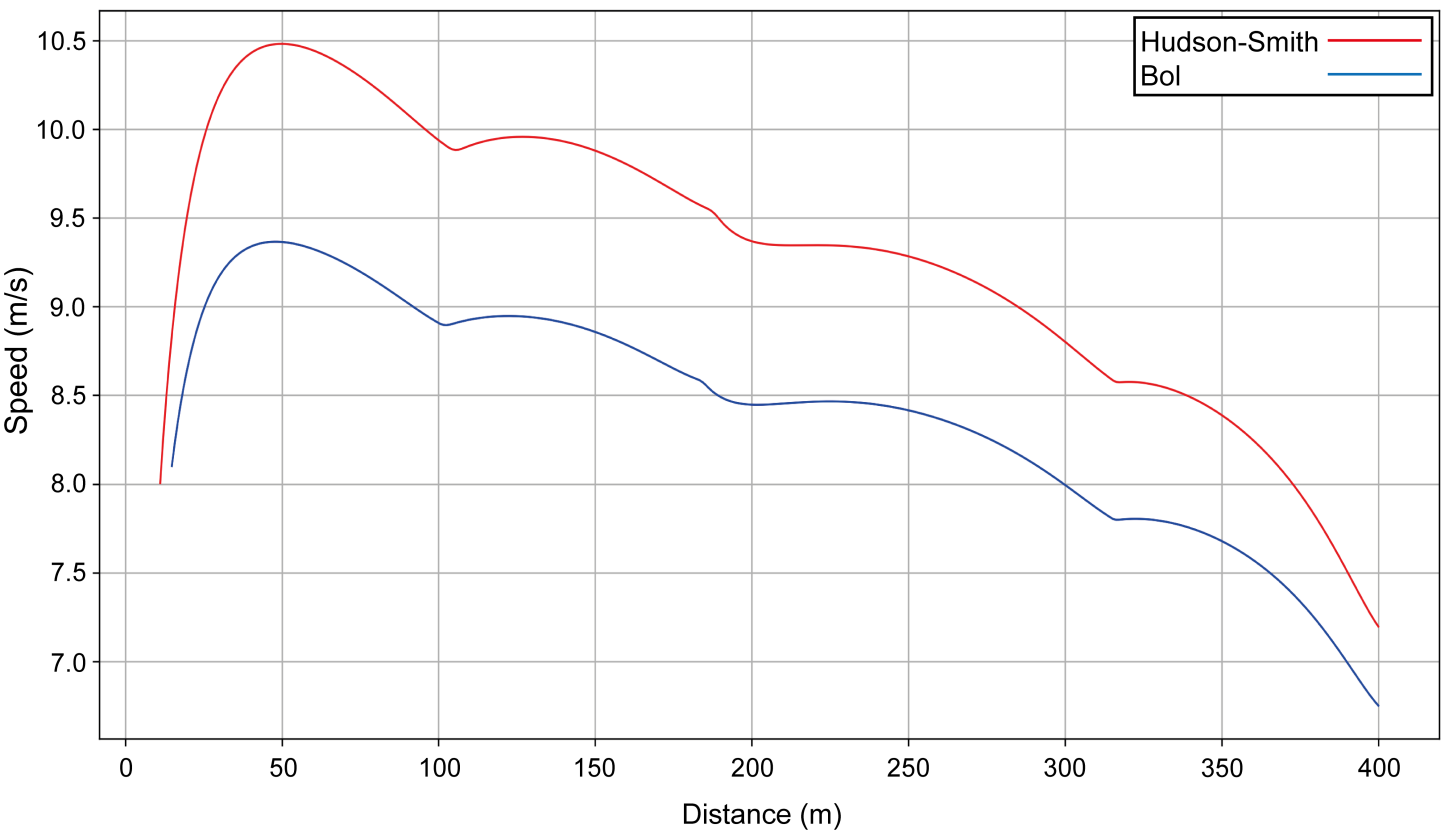
The simulated speed for the winners of the men's (Hudson–Smith, lane 4, red line) and women's (Bol, lane 5, blue line) 400 m finals at the 2022 European Athletics Championships in Munich.

We identify the mathematical parameters of each athlete and produce a simulation of the race; in each case, the computed intermediate times for each 100-m split match the official split times for both men's and women's 400 m and 1,500 m finals (32) with a precision better than 0.1 s. The simulations allow us to precisely see the effect of the straight (slight acceleration) and the effect of the bends on the women's and men's 400 m winners in Munich (Figure 1). Unsurprisingly, Bol runs slower than Hudson–Smith as she has a lower estimated final VO_2_ (53.2 ml/kg/min vs. 69.6 ml/kg/min), but has an anaerobic contribution that is slightly higher (77.6% vs. 76.0%), which explains her smaller absolute decrease in velocity during the last 100 m.

#### 3.1.1 Effect of the parameters

**Figure 2:**
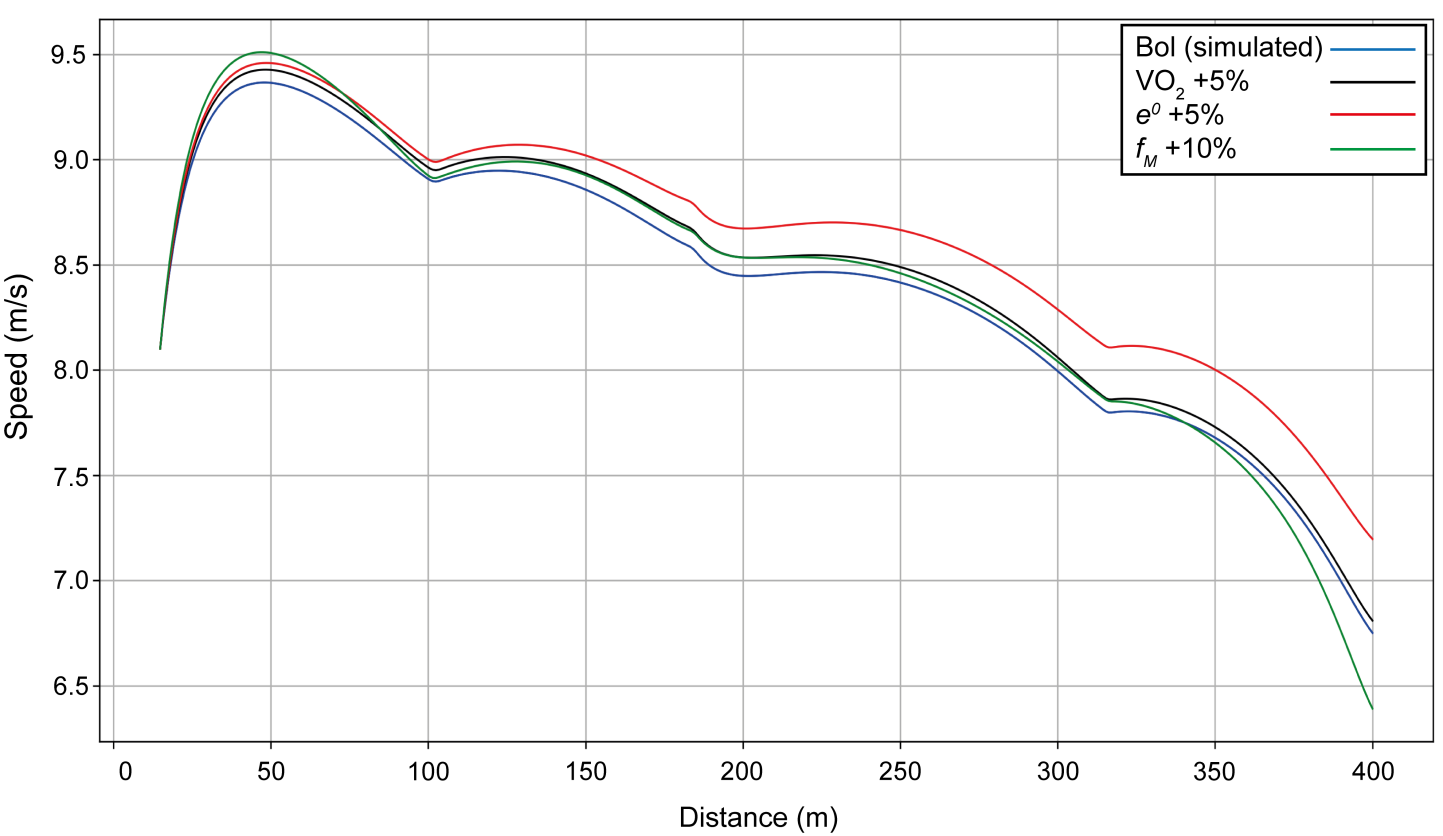
Simulated speed for Bol for her race in Munich (blue line), with the final value of VO_2_ increased by 5% (black line), with anaerobic energy *e^0^* increased by 5% (red line), and with the maximal force *f_M_* increased by 10% (green line).

Using Bol as our example (finishing time: 49.44 s), we include velocity curves for the women's 400 m when anaerobic energy *e^0^*, propulsive force *f_M_*, and maximal value of VO_2_ are varied (Figure 2). When the anaerobic energy *e^0^* is increased by 5% (red line on Figure 2), the whole velocity curve moves upward, and the simulated finishing time is improved (reduced) by 1.2 s. When only the final value of VO_2_ is increased by 5% (black line), the optimal race occurs when the athlete starts faster, but then has a decrease in her velocity because the anaerobic contribution is not sufficient; the end of the race is then similar to having a smaller VO_2_, with a finishing time improved by 0.3 s. When the maximal force *f_M_* is increased by 10% (green line), the start is undertaken with more propulsive force, leading to a much quicker first 100 m, but speed decreases more at the end because there is not enough anaerobic energy left and the overall improvement in finishing time is only 0.1 s.

#### 3.1.2 Effect of the lane

**Figure 3:**
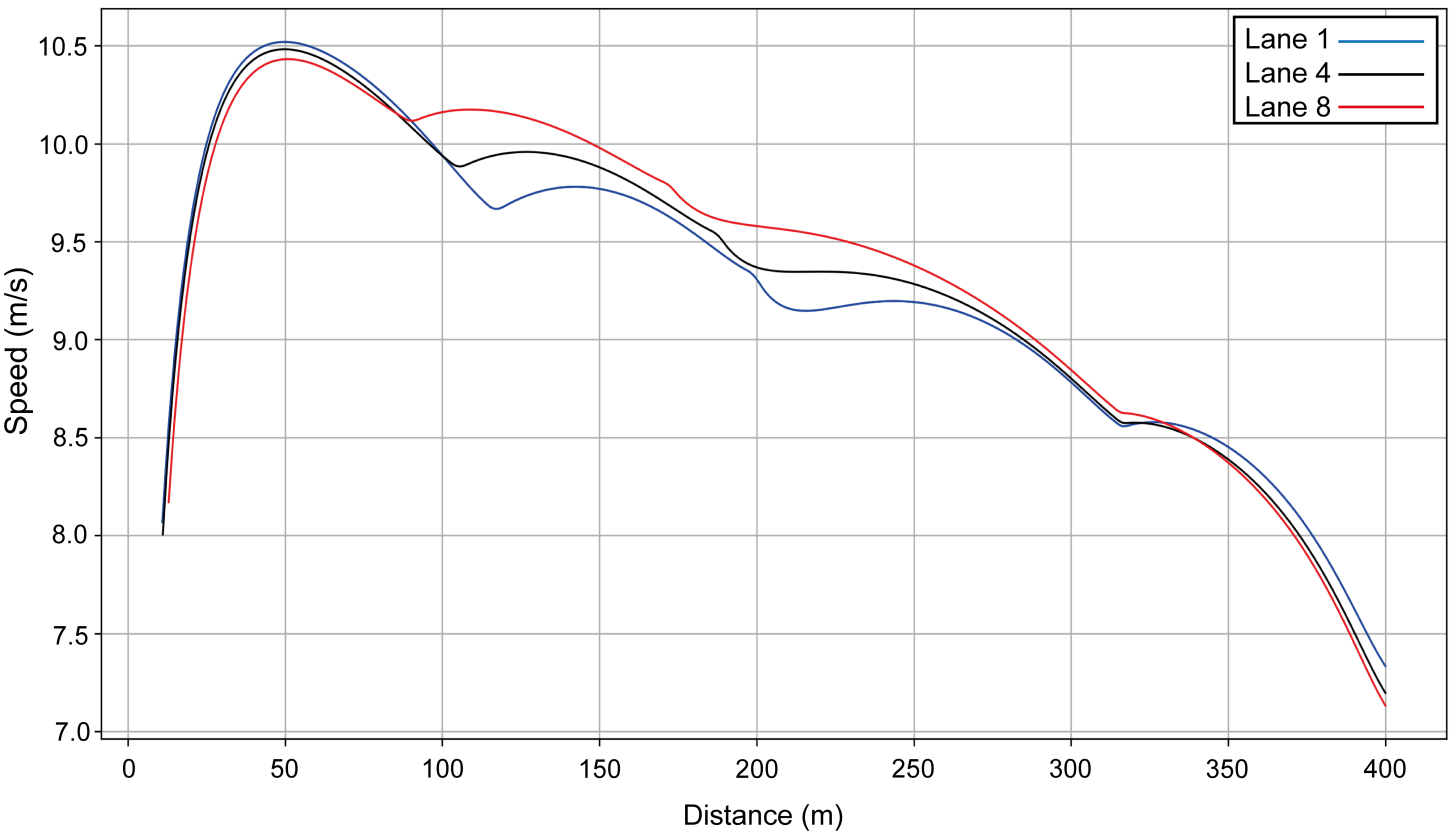
Simulated speed for Hudson–Smith for his race in Munich (lane 4, black line) compared with his same performance simulated for lane 1 (blue line) and lane 8 (red line).

We compare the men's winner with a performance in another lane, i.e., we simulate Hudson–Smith's race (finishing time: 44.53 s) as though he had run in another lane to establish the effect of each lane's geometry (Figure 3). In each model, the other variables (VO_2_, *e^0^*, *f_M_*, etc.) are unchanged. The fastest start is in lane 1 (blue line on Figure 3), and the slowest is in lane 8 (red line). If Hudson–Smith runs in lane 1 (simulated data), he starts quickly, but because of the effort caused by centrifugal forces, finishes 0.06 s slower. In lane 8, the finishing time is almost the same, but he runs much faster on the mostly straight part of the track between 100 m and 200 m.

**Figure 4:**
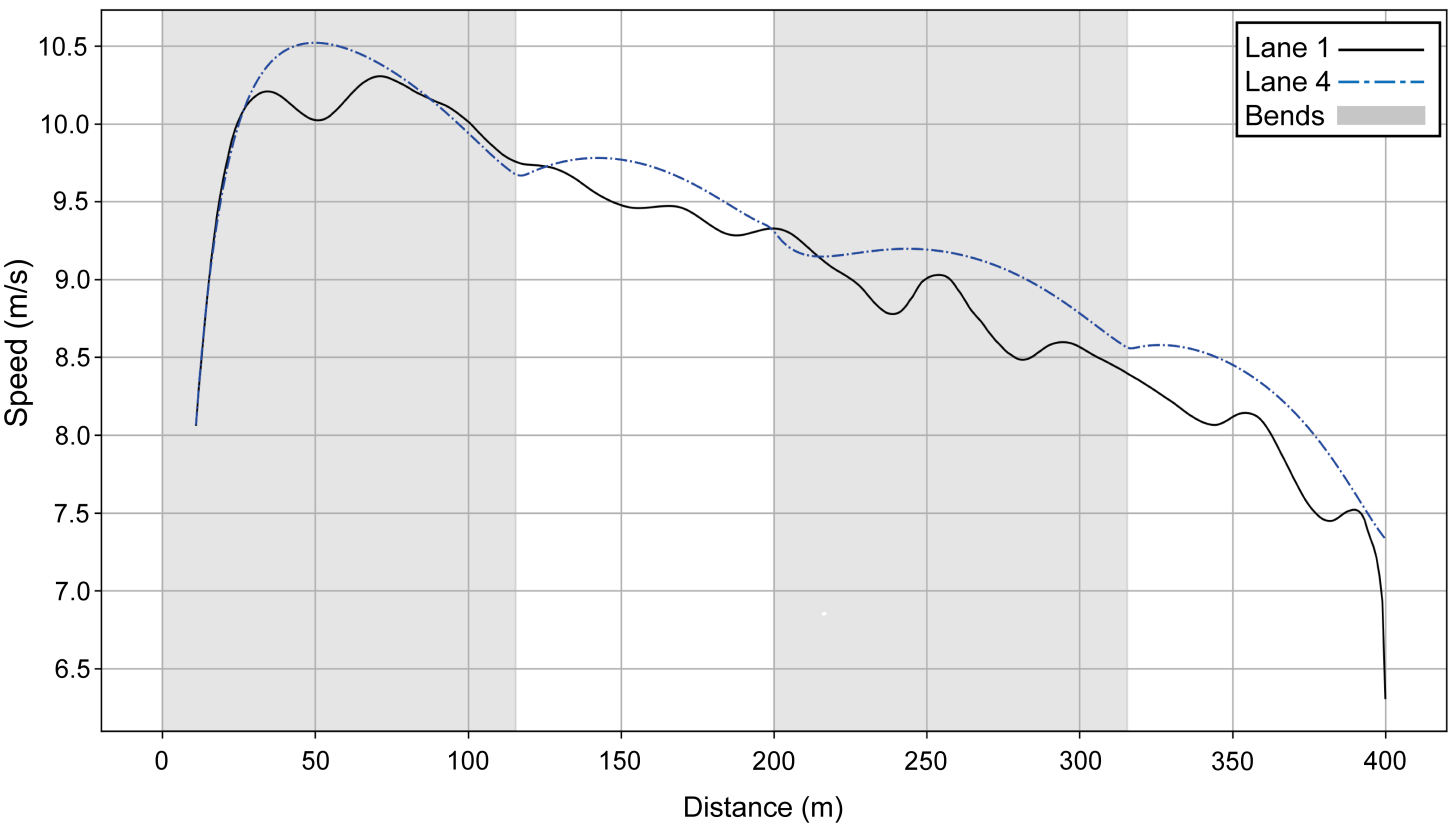
Simulated speed for Hudson–Smith for his race in Munich (blue dashed line) compared with the athlete who competed in lane 1 (black line).

If we compare Hudson–Smith's simulated lane 1 profile with the raw data for the runner who actually ran in lane 1 (Figure 4), we see that they race similarly over the first 200 m. Then, during the second part of the race, the lane 1 athlete loses 0.5 s every 100 m compared with Hudson–Smith (if he had run in lane 1 rather than lane 4).

### 3.2 Description of the 1,500 m races

**Figure 5:**
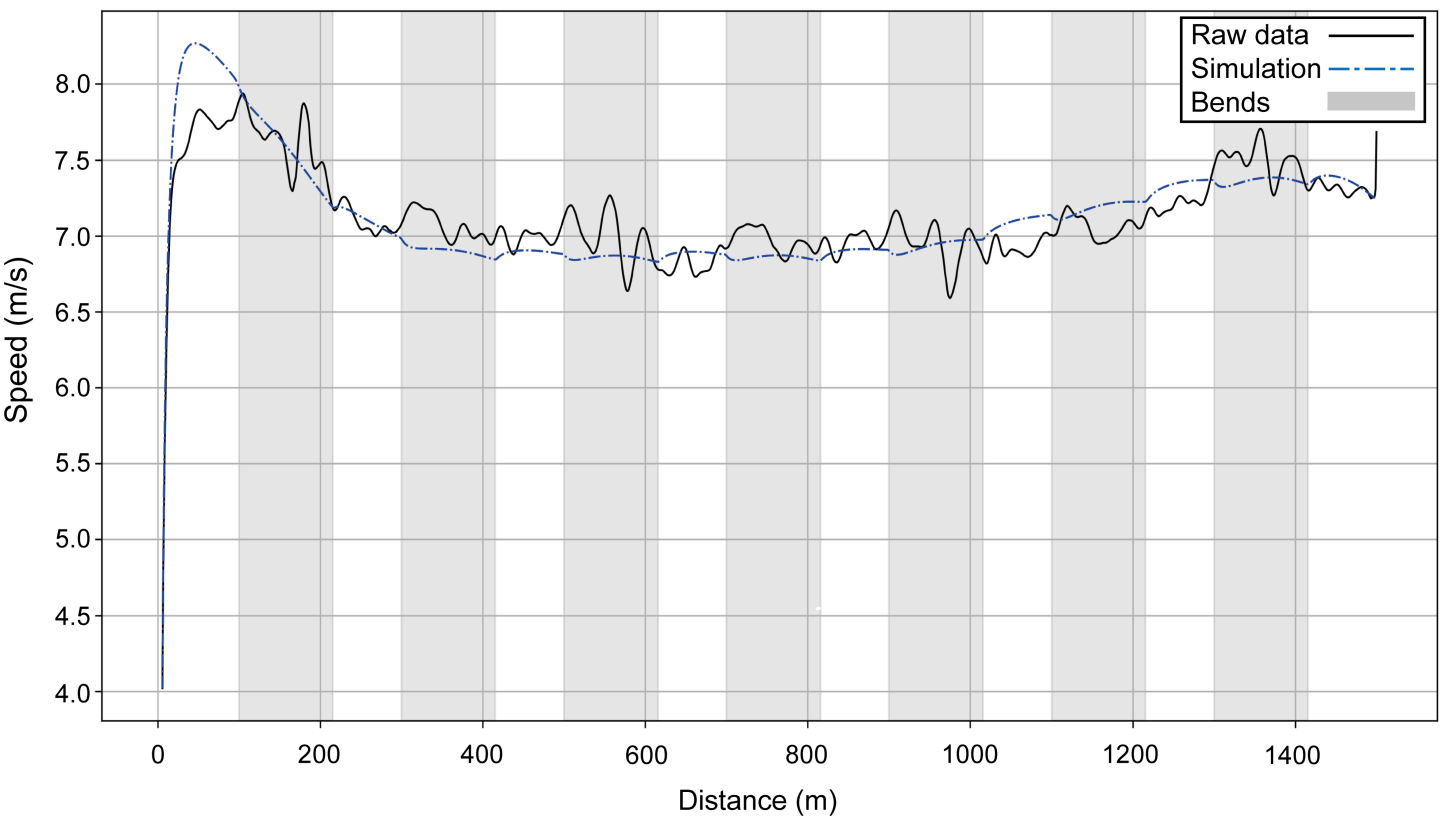
The simulated speed for Ingebrigtsen's performances over 1,500 m at the 2022 European Athletics Championships in Munich (dashed blue line) and the original raw data (black line).

**Figure 6:**
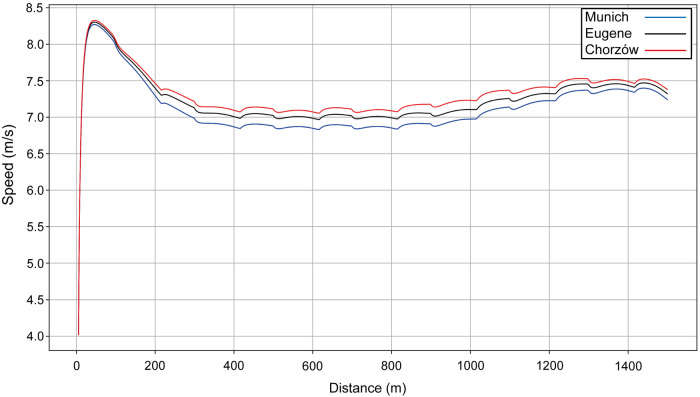
The simulated speed for Ingebrigtsen's performances over 1,500 m at the 2022 European Athletics Championships in Munich (blue line), the 2022 World Championships in Eugene (black line) and the 2023 Diamond League event in Chorzów (red line).

**Figure 7:**
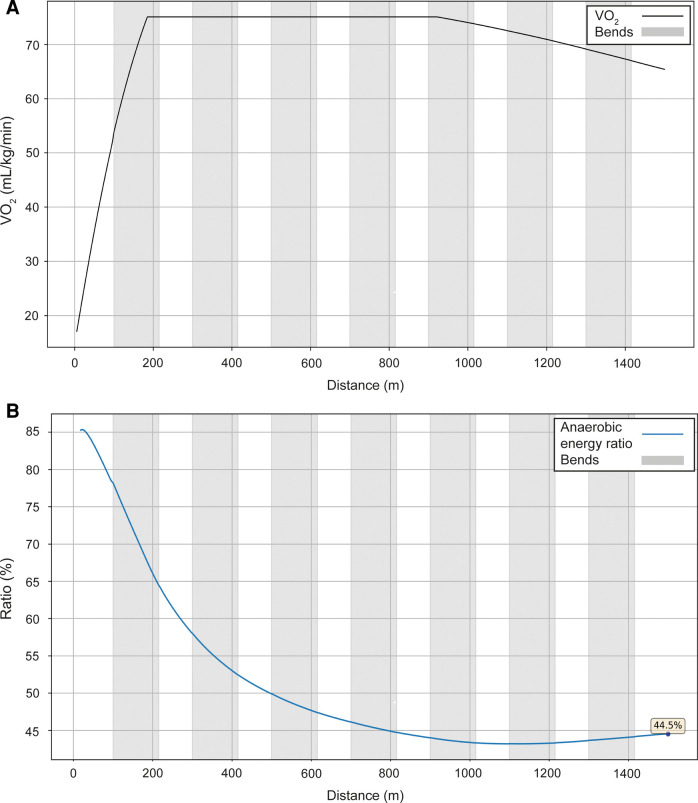
The simulated VO_2_ (**A**) and anaerobic energy to total energy ratio data (**B**) for Ingebrigtsen's performance over 1,500 m at the 2022 European Athletics Championships in Munich.

Next, we present the simulation for Ingebrigtsen's 1,500 m final in Munich (2022 European Championships) (Figure 5): a strong acceleration at the beginning, followed by a cruising speed that is a little slower, and an acceleration at the end. In comparing Ingebrigtsen's race in Munich with his performances in Eugene and Chorzów (Figure 6), we compute an increase in his anaerobic capacity by 5.7% and 10.0%, respectively. The results show a strong agreement between the official split and finishing times obtained by the chronometer and the simulated data in each of these races (Table 1), with a precision to the final time to 0.05 s, and to less than 0.1 s for 80% of the split times. For his Munich race, Ingebrigtsen has a modeled VO_2_ of 78 ml/kg/min and only a 10% decrease in VO_2_ at the end of the race, beginning after ∼800 m (Figure 7A). He has a high cruising speed because of a high VO_2_ and has a high anaerobic energy ratio of 45% (Figure 7B).

**Table 1:** Official and simulated split times for each 100-m race segment from 200 m onwards for Jakob Ingebrigtsen's 1,500 m performances in Munich (2022 European Athletics Championships, 1st place), Eugene (2022 World Athletics Championships, 2nd place), and Chorzów (2023 Diamond League, 1st place and PR). The official split times from the Chorzów race were reported to one decimal place.

Distance (m)	Munich (official)	Munich (simulated)	Eugene (official)	Eugene (simulated)	Chorzów (official)	Chorzów (simulated)
200	0:28.09	0:28.18	0:28.09	0:28.01	0:27.7	0:27.90
300	0:42.20	0:42.22	0:41.86	0:41.81	0:41.8	0:41.54
400	0:56.34	0:56.70	0:55.90	0:55.99	0:55.8	0:55.56
500	1:10.66	1:11.22	1:10.00	1:10.22	1:09.8	1:09.61
600	1:25.08	1:25.80	1:24.17	1:24.52	1:23.8	1:23.73
700	1:39.75	1:40.35	1:37.69	1:38.77	1:37.8	1:37.80
800	1:54.11	1:54.92	1:52.04	1:53.06	1:51.6	1:51.91
900	2:08.52	2:09.43	2:06.12	2:07.28	2:05.5	2:05.90
1,000	2:23.04	2:23.86	2:20.28	2:21.43	2:19.3	2:19.81
1,100	2:37.51	2:38.00	2:34.23	2:35.32	2:33.1	2:33.47
1,200	2:51.68	2:51.94	2:48.28	2:49.06	2:46.9	2:47.03
1,300	3:05.62	3:05.60	3:02.13	3:02.56	3:00.4	3:00.38
1,400	3:19.08	3:19.19	3:15.69	3:16.00	3:13.8	3:13.72
1,500	3:32.76	3:32.79	3:29.47	3:29.47	3:27.14	3:27.09

#### 3.2.1 Effect of the parameters

**Figure 8:**
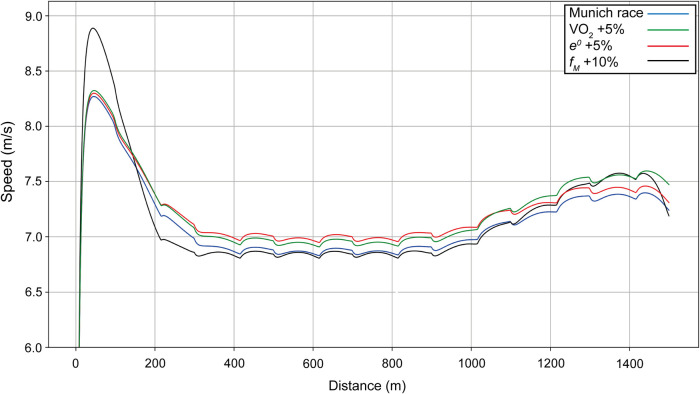
Simulated speed for Ingebrigtsen for his race in Munich (blue line), with the final value of VO_2_ increased by 5% (green line), with anaerobic energy *e^0^* increased by 5% (red line), and with the maximal force *f_M_* increased by 10% (black line).

When Ingebrigtsen's anaerobic capacity is increased by 5% (Figure 8, red line), this results in an increase in cruising speed during the race, although not at the beginning, for a simulated improvement in finishing time of 2.86 s. In his Chorzów PR race, we compute that he has increased his anaerobic capacity by 10%, which allowed a 5-s improvement in finishing time. Increasing the maximal force (black line) changes the initial acceleration and the final sprint, but reduces the cruising speed with a net improvement of 0.55 s. When VO_2_ is increased by 5% (green line), the cruise velocity increases, but not as much as with an increase in anaerobic capacity; since the difference between the maximal and final value of VO_2_ also increases, the final velocity is higher than for an increase in anaerobic capacity for an overall improvement in finishing time of 2.98 s.

**Figure 9:**
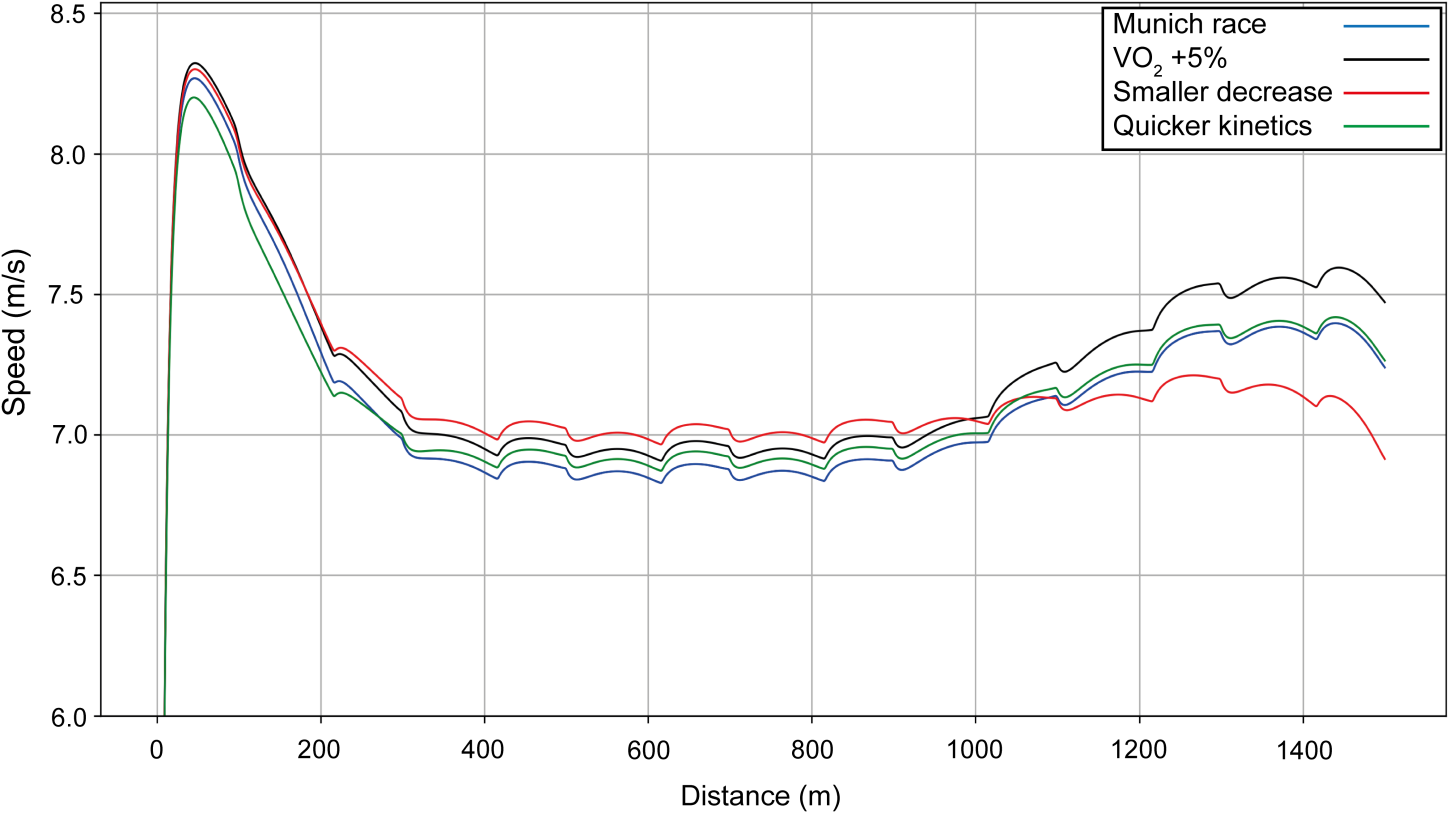
Simulated speed for Ingebrigtsen for his race in Munich (blue line), with the final value of VO_2_ increased by 5% (black line), with a smaller decrease in VO_2_ toward the end of the race (red line), and with quicker VO_2_ kinetics (green line).

We also simulate varying VO_2_ values and oxygen kinetics for Ingebrigtsen (Figure 9): we increase the VO_2_ kinetics (green line) so that the athlete reaches his maximal value of VO_2_ quicker, and he requires a smaller initial acceleration. We see that the optimal strategy is to start with a peak velocity approximately 0.07 m/s slower, which allows for a higher cruising speed; the overall improvement in finishing time is 0.49 s. Thus, the model indicates that it is likely Ingebrigtsen has faster VO_2_ kinetics than his opponents and this is why he starts a little slower. If the VO_2_ has a smaller decrease at the end (red line) (VO_2_ is kept almost constant), then the cruising velocity is faster and the final acceleration is smaller, and the net improvement in finishing time is 1.15 s.

#### 3.2.2 Comparison between U23 and senior performances

**Figure 10:**
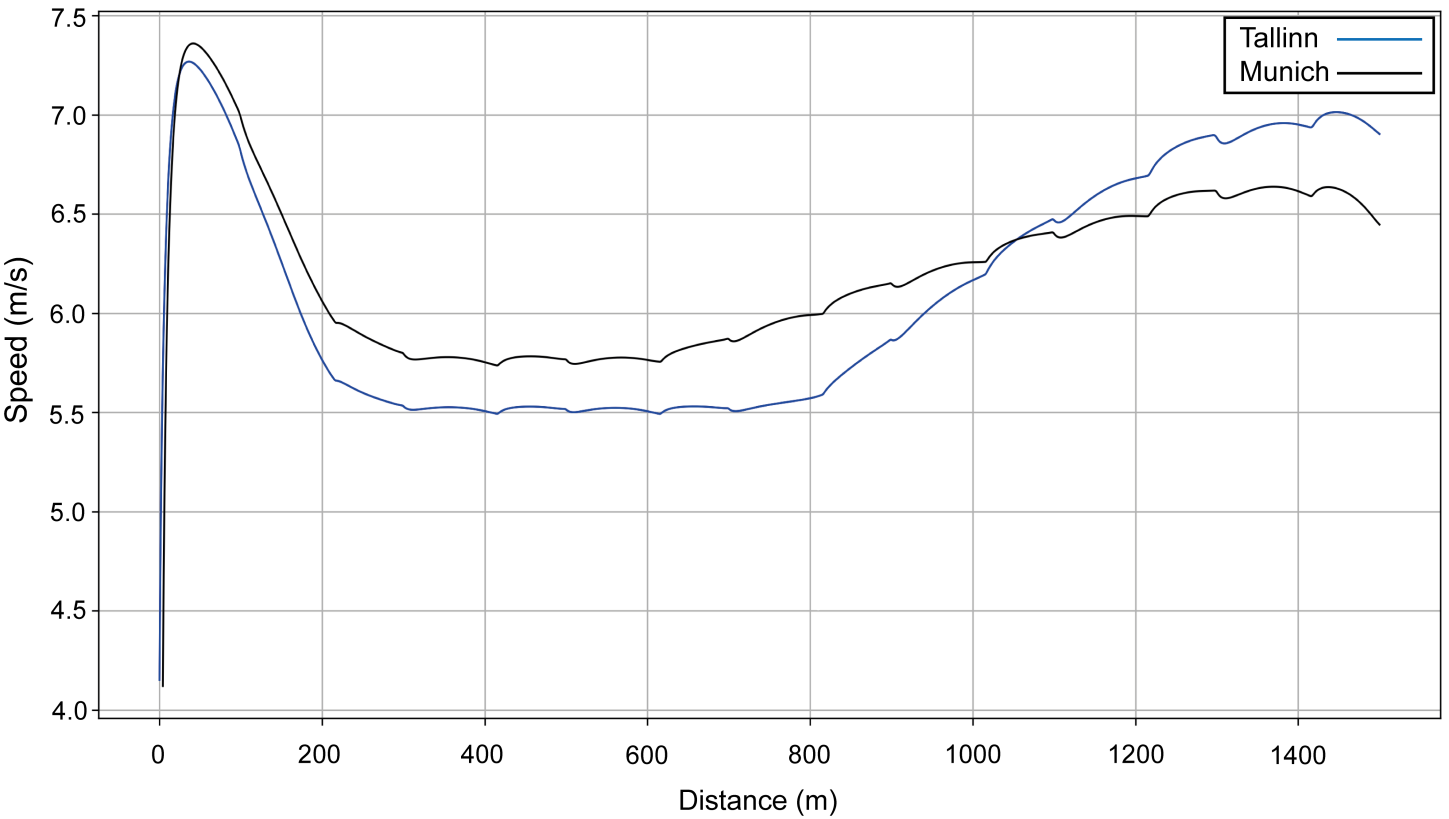
The simulated speed for Sabbatini's performances over 1,500 m at the European U23 Athletics Championships in Tallinn in 2021 (blue line) and the 2022 European Athletics Championships in Munich (black line).

**Figure 11:**
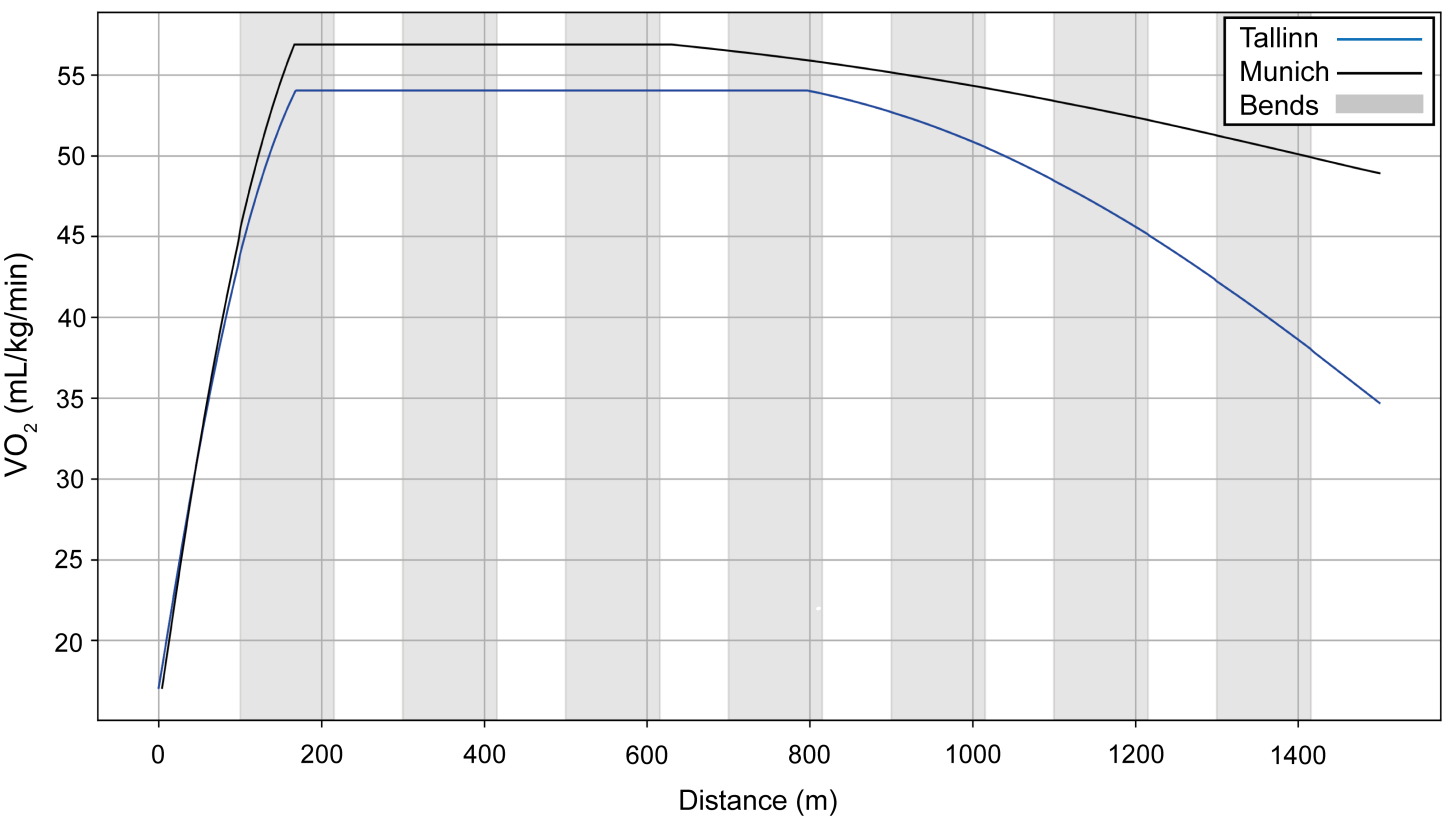
The simulated VO_2_ data for Sabbatini's performances over 1,500 m at the 2021 European U23 Athletics Championships in Tallinn and the 2022 European Athletics Championships in Munich.

**Figure 12:**
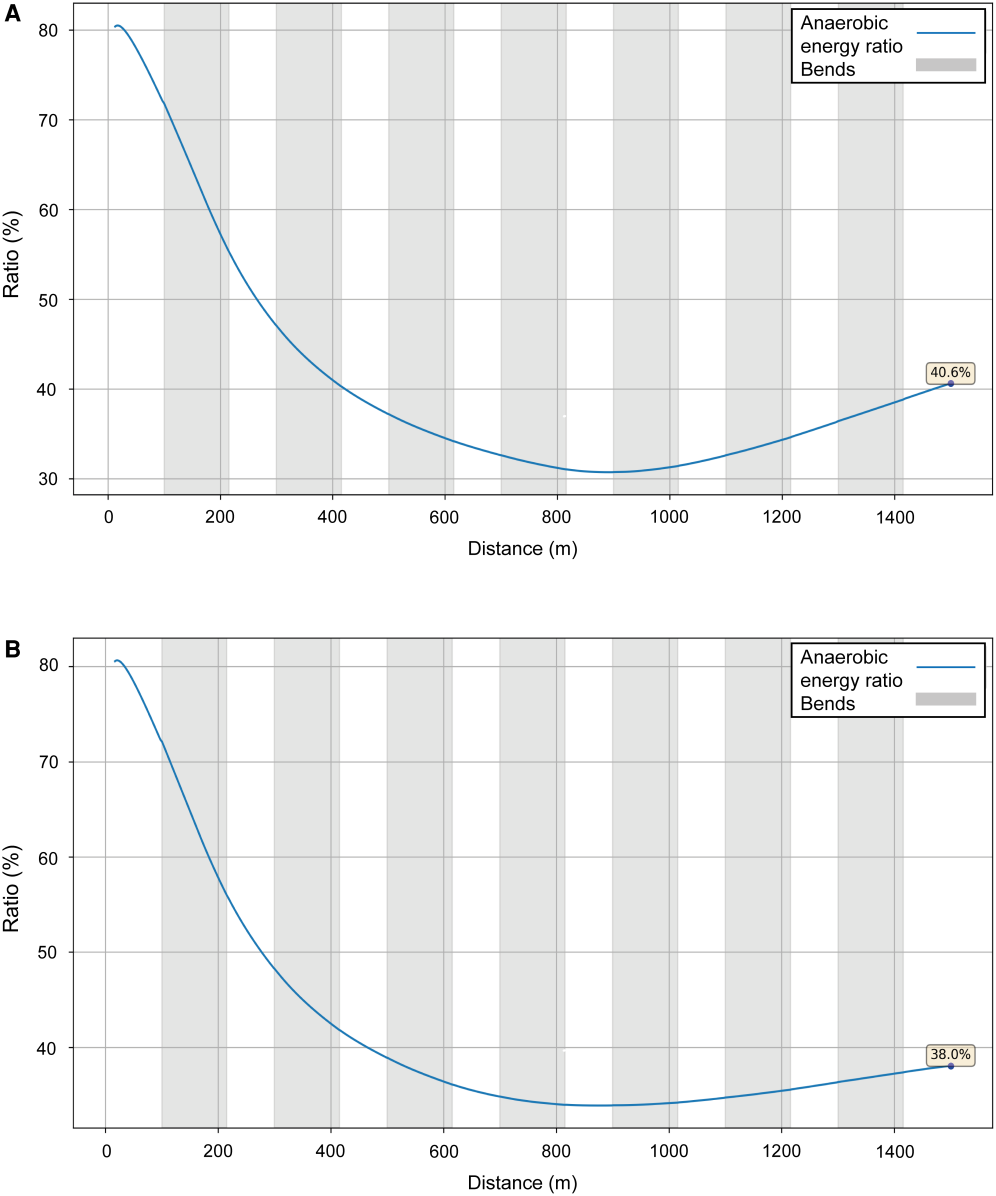
The simulated anaerobic energy to total energy ratio for Sabbatini's performances over 1,500 m at the 2021 European U23 Athletics Championships in Tallinn (**A**) and the 2022 European Athletics Championships in Munich (**B**).

We model two performances for Sabbatini (Figure 10): in the U23 Championships, her VO_2_ dropped sharply after 800 m (Figure 11, blue line), which is why she relies more on anaerobic energy for the second part of the race and speeds up when VO_2_ starts to decrease. In the simulation, her VO_2_ decreases gradually from roughly 54 ml/kg/min in the middle of the race, where her running speed is ∼5.5 m/s, down to 36 ml/kg/min by the very end of the race, where her speed is ∼7 m/s (Figure 10). A year later, in Munich, she managed to maintain her VO_2_, with a decrease of only 10% at the end (Figure 11, black line), so she did not accelerate as much in the second half and could run at a higher pace (closer to 6 m/s rather than 5.5 m/s). Sabbatini's overall anaerobic ratio is ∼40% in Tallinn (Figure 12A) and 38% in Munich (Figure 12B), but in Tallinn the anaerobic ratio drops to 30% in the middle of the race.

## 4 Discussion

The aim of this study is to model World-class racing performances to allow for a deeper understanding of the factors that affect running speed and to establish the effect of the bends. Using the model, we are able to show that both the men's and women's 2022 400 m European Champions run with a fast start, where peak running velocity is achieved as early as 50 m. These particular athletes then progressively slow until the end of the race, but with an effect of the bends manifesting through slight decelerations into, and slight accelerations out of, each bend. Using our modeled data of two athletes (including the winner) from the men's 400 m race, we see that the smaller radius of the inside lane results in worse performances than a middle lane. The runner who ran in lane 1 (Figure 4) manages to start as fast as Hudson–Smith (if he had run in lane 1), probably because the temptation to follow his opponents who are visibly in front helps him in this fast start. Nevertheless, our modeling shows that he has a lower anaerobic energy, and therefore cannot maintain his speed, compared with what Hudson–Smith would have performed in lane 1.

The effect of the lanes is generally well understood by coaches (8) and, as the middle lanes in the semi-finals and final are allocated to the highest placing athletes in the previous round (33), our findings emphasize the need for athletes to run quickly enough in qualifying to achieve a top qualifying position (34). There is less reason to worry about an outside lane draw from the perspective of bend radius and its effects on running mechanics, and indeed the larger radius could be of advantage: van Niekerk (RSA) set the World Record during the 2016 Olympic Games in the outermost lane (8). However, there could be a psychological disadvantage to being less able to pace oneself using other athletes in lane 8, which coaches should note when preparing athletes for competition. Those tracks that use nine lanes rather than eight, and where the 400 m athletes are allocated lanes 2–9 (33), are better suited to faster running.

The 400 m race is an event that relies on important contributions from both the aerobic and anaerobic energy systems. Our simulations show that the anaerobic component is ∼76%–77% for both male and female European 400 m champions, which aligns with previous studies on similar-standard athletes using post-race blood lactate measurements (16) and mathematical modeling (35). In general, the longer the run duration, the greater the aerobic contribution, and this might explain why previous research (4) on slower 400 m athletes (mean best times for men: 52.2 s; women: 60.2 s) had anaerobic components averaging less than 60%. Our modeling clearly shows that anaerobic contributions smaller than ∼76% inevitably mean slower times, and reiterates the recommendation for coaches to prioritize anaerobic training in preparation for 400 m racing. The biggest improvement in finishing time arises when anaerobic energy is modeled to be 5% greater, much more so than increasing VO_2_ or maximal force. Indeed, we see that applying a high force at the beginning of the race leads to a strong acceleration during the first bend, but this is ultimately counterproductive as an insufficiently high anaerobic capacity means only that the eventual deceleration is considerably worsened. Nevertheless, although the total aerobic contribution to 400 m performance is estimated to be only ∼24%, the importance of the role of a high fractional utilization of VO_2_ max is seen. Indeed, the higher the VO_2_, the smaller the decrease in velocity toward the end of the race, although a high anaerobic energy is crucial here as well.

It is worth confirming previous findings that a fast start is essential for a quick finishing time (36); when we simulate a faster start for Bol (by increasing *f_M_* by 5%), a slightly quicker finishing time is achieved. This highlights the role of a fast start in optimizing oxygen uptake kinetics, in that the athlete will undergo oxygen deficit during the early stages of exercise (over any race distance), and reducing this deficit is a key factor in performance (37). Although it could seem sensible to start the race slower and try to have a more even pace, the short duration of the 400 m event does not permit the athlete to benefit enough from any anaerobic energy spared during the first 200 m. For one thing, the inability of an athlete to catch up lost time on their rivals means that this is an ineffective tactic (38) and, in any case, trained 400 m athletes are able to reach a high fractional utilization of VO_2_ max less than 25 s from the start (39). The 400 m race is best described not as an all-out pacing profile but a controlled fast pace with a strategy of minimizing deceleration during the latter, aerobic-dominant stages, which requires adequate development in training. It should be noted that Bol is the 2023 World Champion over the 400 m hurdles, and the longer duration of the hurdles event requires a larger aerobic contribution (40); this training and racing background might have helped her maintain pace better during the second half of the flat 400 m. We should also note that in the 400 m event, although the best athletes like Bol appear to be speeding up toward the end, the actual cause of the increase in distance between athletes is due to how much more the less successful athletes slow during the last 100 m.

Although it is a longer and more aerobically dominant event, success in the 1,500 m race also depends on the effective use of anaerobic resources (41). The typical pacing profile adopted in championship 1,500 m racing is one where there is a fast opening 100 m (2), which can aid oxygen uptake kinetics (42), and is important in getting into or close to the inside lane on the first bend for tactical reasons; indeed, the tactical choices made by athletes can have an effect on the pacing profiles that occur, especially in the early part of the race (notwithstanding that these are strongly influenced by an athlete's physiological capacity). The early fast start along the back straight is evident in our race simulations for both analyzed male and female athletes in Munich, although of course these speeds are much slower than what are found in the 400 m events: in the men's race by ∼2.5 m/s, and in the women's by ∼2 m/s. As in the 400 m, there is a negative effect of the bends shown by the velocity profiles and, aside from the negative effect on running mechanics, obtaining a position near the kerb is a sensible tactic throughout the race to reduce total distance run (43). Peak velocity during the fast start occurs after ∼50 m and, indeed, is the fastest the athletes run during the entire race. A considerable deceleration then occurs until 200 m, after which a smaller reduction of speed continues to 300 m (i.e., before the second bend). The athletes then enter a “cruising” phase, where pace is kept relatively steady before being gradually increased from 800 m until the sprint finish (last 200–300 m).

The pacing profile adopted by 1,500 m athletes in championship racing is generally known as J-shaped pacing (1), but this is demonstrated more obviously in the women's example than the men's. Those athletes whose VO_2_ decreases in the second part of the race cannot maintain a high cruising speed if they want to perform their optimal race, which is to speed up in the last two laps. If, by contrast, they follow a pace run at a high cruising speed, they are then unable to keep up in the last two laps. In the men's race, Ingebrigtsen's pace is more even than in typical championship 1,500 m finals (6) and the simulation shows that this is related to his very high VO_2_ that he is able to maintain throughout the race. In this regard, Ingebrigtsen's modeled VO_2_ during the race (78 ml/kg/min) reflects well the maximal aerobic output reported in previous literature on elite male 1,500 m runners (44), including Jakob Ingebrigtsen's older brother and 2012 European Champion, Henrik, whose VO_2_ max was reported from laboratory testing as 84.4 ml/kg/min (45). He probably has very fast VO_2_ kinetics that allows him to start not as fast as his opponents and save anaerobic energy. What this means in effect is that Ingebrigtsen is able to maintain a very fast pace, but which uses anaerobic energy on top of a substantial aerobic contribution. The values for Ingebrigtsen are substantially higher than have been reported in previous research (41); however, as in the case of the 400 m, this is partly because the non-elite athletes previously studied took much longer to complete the 1,500 m distance, and the slower the athlete, the more they rely on aerobic energy contributions. In our models, the anaerobic contribution is greater than the aerobic contribution during the fast start, as occurs over all race distances where the exercise domain is heavy or severe (42), and during the sprint finish. However, in the case of Ingebrigtsen, anaerobic energy is likely depleted in a linear fashion from start to finish, meaning that there is only a slight increase in speed of ∼0.40 m/s over the last 500 m; by comparison, the typical increase during this last third of the race is ∼0.75 m/s in men's championship racing (6). Ingebrigtsen therefore adopts a fast pace throughout the race, leading from 300 m to the end, which is dependent on a continuously high energy provision via both aerobic and anaerobic resources, and that presumably is intended to deplete rivals' anaerobic stores so that the race is effectively won before the sprint finish even begins. Such a racing strategy clearly depends on the development of both the aerobic and anaerobic systems to a great extent, and where this racing approach works, as in this race in Munich and in the Olympic Games (46), the reward is a gold medal; however, the disadvantage of this tactic is that other athletes who can maintain contact and have faster sprint finishes can prevail, as in the 2022 and 2023 World Championships finals (47, 48). Ingebrigtsen also won the 5,000 m race at the 2022 European Championships in Munich and at the World Championships three weeks beforehand (49), an event that relies even more on a high fractional utilization of a high VO_2_ max (44). This emphasis on the longer range of middle-distance racing suggests a “1,500–5,000 m type” runner (50) and explains well his approach of starting slower than others and running at a higher cruising speed in the middle part of the race. In essence, because we calculate that Ingebrigtsen has a very high VO_2_, the capacity to maintain this VO_2_ and fast oxygen uptake kinetics, he does not start the race as fast as others, which could unintentionally provide a competitive advantage in avoiding the crowding that occurs before the first bend, and can cruise during the middle part of the race at a high speed, as in his PR race in Chorzów. By contrast, if rival athletes do not have the same VO_2_ characteristics and attempt to cruise at the same speed, they are likely to be unable to accelerate in the later stages of the race.

We choose to analyze an athlete in the women's race, Sabbatini, who was not the eventual winner but had also competed in (and won) the European U23 competition in Tallinn the previous year with quite different pacing profiles, and for which we could model similar high-resolution pacing data. Although her finishing position in Munich was lower than in Tallinn, her finishing time was quicker by nearly 8 s. The simulations for each race show that the deciding factor in her quicker run is an increased VO_2_ throughout the race (both in terms of its maximum and end values), bearing in mind that she might have not needed to fully exert herself in winning the U23 event. The effect of this increased VO_2_ is seen in her cruising speed between 300 and 800 m, which is ∼0.3 m/s higher in Munich. There is no difference in starting speed (related to *f_M_*), although her sprint finish is slower in Munich than in Tallinn, and might result from an earlier depletion of anaerobic energy in maintaining pace with her opponents (she was always in the top five positions until 1,400 m). This would have occurred as the higher mean force required to maintain a higher running speed is dependent on anaerobic energy and shows that her faster cruising speed (compared with Tallinn) did not just arise from an improved VO_2_; as in most 1,500 m races, this is because running speed was greater than the velocity at VO_2_ max (44, 51). Unlike in the men's race, the depletion of anaerobic reserves is not linear because the slower section in the first third of the race reduces the rate of anaerobic energy use. As with the winner of the men's race, there is a sound reason for adopting a faster pace in the middle section of the race to obtain a quicker finishing time, but which does reduce the potential for a strong sprint finish.

We show in our examples of both men's and women's 1,500 m racing that there is a contrast between relying on a constant but fast pace and the less optimal approach of running slower but maintaining a better capacity for a fast sprint finish. Given there will be a natural limit on how much training an athlete can complete, and how much the human body can improve any particular variable (52, 53), we run simulations on Ingebrigtsen's race to see the effects of increasing VO_2_ vs. increasing anaerobic energy. The models show important improvements of ∼3 s in each scenario, and it is clear that, provided the athlete has a considerably high anaerobic capacity to begin with, there is an advantage to developing the aerobic system for middle-distance running (or vice versa). One difference between his winning performance in Munich and his PR is the presence of other runners to act as pacemakers in the quicker race, who set an even pace and reduce the psychological load and the energy required to maintain the same speed. This reduction in energy usage until at least the middle of the race, when many pacemakers drop out, clearly helps spare anaerobic energy so that it can be used to maintain a high running speed to the end. As with the example from Munich, record attempts require maintaining a relatively even pace that use a constant high energy contribution from both aerobic and anaerobic sources, but which prevent a fast sprint finish.

In this study, we have been able to simulate World-class athlete performances in the 400 m and 1,500 m events. Importantly, the computations show that the proportion of anaerobic energy that is needed to achieve World-class running times in these specific athletes is generally higher than has been previously reported. The main contributors to this difference are the modeling approach taken, the much higher standard of performance being analyzed, and the use of in-competition data rather than laboratory measurements. Some of the results we report could be considered controversial but should be understood as being accurate in the context of mathematical modeling, and do not contradict the meaningfulness of measures calculated in other contexts (e.g., *in vivo* testing). It is beyond the scope of this study to examine the best training practices to achieve higher anaerobic capacity, higher VO_2_ max scores, lower running economy, or any other parameter, but coaches and athletes should nonetheless take our simulations into account when considering the relative value of increasing any particular variable. We show that bend running has a negative effect on running speed, which demands a training approach that helps the athlete accommodate the mechanical requirements of negotiating the turns. Athletes competing over 400 m do not have the luxury of directly choosing their lane, but there is certainly an effect of the inside lane that coaches should note needs training for (especially considering that much of the 4 × 400 m relay is run in the inside lane). Being able to base our predictions and simulations on very high resolution pacing data means that we can account more realistically for changes in pace than traditional 50-m or 100-m split times and mean that more precise calculations of energy contributions are possible. We choose a small range of performances to model that we use to understand different race scenarios (e.g., inside vs. outside lane, faster or slower start) that have practical applications for coaches. These show the need for specialist bend-running training, athlete-specific development of aerobic and anaerobic capacity (in both 400 m and 1,500 m), and the effects of adopting a fast cruising pace throughout the 1,500 m, in particular. We chose here to focus on one athlete per event as this is an efficient approach to undertake when explaining the effect of different factors (anaerobic capacity, running lane, etc.). For each athlete in any event, we could perform an identification of parameters to get a velocity curve close to the race data. However, performing it for each athlete is not a method of corroborating the model since it will always uncover the parameters. Although the quantity of athletes analyzed here might appear small in terms of participant numbers, the concept of “sample size” differs for modelling studies from those for experimental or observational studies commonly conducted in sport and exercise science, and precludes the necessity for multiple analyses. Nonetheless, as well as modeling performances over other distances, future research using this modeling approach could analyze the pacing profiles of close rivals over a range of running distances to assist coaches in understanding the best racing strategies.

## 5 Conclusions

We undertook simulations of 400 m and 1,500 m races using high resolution pacing data (10 Hz) to explain the role of physiological factors, bends, and tactics in relation to successful racing. We show that a fast start is essential in the 400 m and, although this leads to an inevitable slowing throughout the race, is the best strategy. Minimizing deceleration throughout the race relies both on a high anaerobic capacity and a high VO_2_. The lane draw is worst in the inside lane, but an outside lane does not negatively affect performance more so than the middle lanes. We see in the 1,500 m that a capacity to maintain a high VO_2_ throughout the race allows athletes to have a high cruising speed, which requires a large energy contribution from anaerobic resources and does not permit a strong acceleration during the sprint finish. We also note that better performances in the 1,500 m are achieved with higher fractional utilization of a high VO_2_. Coaches need to consider their athletes' physiological strengths when choosing training regimens as some will be better suited to fast, even-paced races that are commonly used in athletics meets (such as the Diamond League, which incorporates pacemaking), and other athletes to the typically variable pacing of championship racing.

## Data Availability

The original contributions presented in the study are included in the article, further inquiries can be directed to the corresponding author.
